# Prevalence and Clinical Implications of Incidental Aortic Arch Abnormalities on Contrast-Enhanced Neck MR Angiography: A Single-Center Experience

**DOI:** 10.3390/medicina59061172

**Published:** 2023-06-19

**Authors:** Minhee Hwang, Dae-Seob Choi, Kwang-Ho Choi, Hye-Jin Baek, Eun Cho, Jong-Myung Park

**Affiliations:** 1 Department of Medicine, Gyeongsang National University College of Medicine, 816-15 Jinju-daero, Jinju 52727, Republic of Korea; hmh8807@naver.com (M.H.); sartre81@gmail.com (H.-J.B.); sgeisilver@naver.com (E.C.); 2 Department of Radiology, Biomedical Research Institute, Pusan National University Hospital, Pusan National University School of Medicine, 179 Gudeok-ro, Seo-gu, Busan 49241, Republic of Korea; 3 Department of Radiology, Gyeongsang National University Hospital, Gyeongsang National University School of Medicine, 79 Gangnam-ro, Jinju 52727, Republic of Korea; 4 Department of Thoracic and Cardiovascular Surgery, Research Institute for Convergence of Biomedical Science and Technology, Pusan National University Yangsan Hospital, Pusan National University School of Medicine, 20 Geumo-ro, Mulgeum-eup, Yangsan-si 50612, Republic of Korea; 5 Department of Radiology, Gyeongsang National University Changwon Hospital, Gyeongsang National University School of Medicine, 11 Samjeongja-ro, Seongsan-gu, Changwon 51472, Republic of Korea; 6 Department of Thoracic and Cardiovascular Surgery, Busan Medical Center, 359 World cup-daero, Yeonje-gu, Busan 47527, Republic of Korea; bulnamu@gmail.com

**Keywords:** aortic arch, incidental aortic arch lesions, aortic arch abnormalities, neck, magnetic resonance angiography

## Abstract

*Background and Objectives*: Vascular abnormalities within the anatomical coverage are frequently encountered in imaging studies. The aortic arch is often overlooked as an anatomical blind spot, especially in neck magnetic resonance (MR) angiography. This study investigated the prevalence of incidental aortic arch abnormalities. We also estimated the potential clinical significance of aortic arch abnormalities as blind spots detected on contrast-enhanced neck MR angiography. *Materials and Methods*: Between February 2016 and March 2023, 348 patients were identified based on contrast-enhanced neck MR angiography reports. The clinical and radiological characteristics of the patients and the presence of additional imaging studies were assessed. The aortic arch abnormalities and coexisting non-aortic arterial abnormalities were classified into two categories according to their clinical significance. We performed the χ^2^ test and Fisher’s exact test for group comparisons. *Results*: Of the 348 study patients, only 29 (8.3%) had clinically significant incidental aortic arch abnormalities. Among these 348 patients, 250 (71.8%) and 136 (39%) had intracranial and extracranial abnormalities, respectively; the clinically significant intracranial abnormalities in the two groups were 130 lesions (52.0%) and 38 lesions (27.9%), respectively. In addition, there was a significantly higher tendency of clinically significant aortic arch abnormalities (13/29, 44.8%) in the patients who had clinically significant coexisting non-aortic arterial abnormalities than in the other group (87/319, 27.3%) (*p* = 0.044). The patient groups with clinically significant intracranial or extracranial arterial abnormalities had higher rates of clinically significant aortic abnormalities (31.0% and 17.2%), but there was no statistical significance (*p* = 0.136). *Conclusions*: The incidence of clinically significant aortic arch abnormalities was 8.3% on neck MR angiography, with a significant association between aortic and coexisting non-aortic arterial abnormalities. The findings of this study could improve the understanding of incidental aortic arch lesions on neck MR angiography, which is of crucial clinical importance for radiologists to achieve accurate diagnoses and management.

## 1. Introduction

In daily clinical practice, radiologists frequently encounter incidental findings within the anatomical coverage in all imaging modalities [1,2,3]. The detection of incidental findings has increased over time due to the escalating need for precise diagnostic imaging as well as the rapid advancements in technology and diagnostic efficacy of imaging modalities in radiology [4,5,6]. In particular, there has been a sharp rise in the prevalence of neck magnetic resonance (MR) angiography imaging, which is a powerful noninvasive technique, used to diagnose various cervical and intracranial arterial diseases [7]. Thus, radiologists may increasingly encounter incidental aortic arch abnormalities within the anatomical coverage of neck MR angiography. However, the evaluation of the incidental aortic arch lesions detected on neck MR angiography has some limitations. This is because neck MR angiography mainly focuses on assessing the origin sites and entire courses of major neck arteries in its scan range despite also covering the aortic arch in the scan range. In addition, the aortic arch frequently turns into an anatomical blind spot on neck MR angiography due to the following factors: partial or non-optimal visualization of the aortic arch because of the patient’s respiratory or motion artifacts, inadequate coverage related to scan range differences among hospitals, or non-availability of synchronous dedicated additional sequences for the incidental aortic arch abnormalities during the examination [8,9].

The aortic arch, which is located between the ascending and descending aorta, has three main branches, including the brachiocephalic trunk, the left common carotid artery, and the left subclavian artery [10]. Abnormalities of the aortic arch can be accompanied by a wide range of lesions, including both physically normal and clinically significant varieties. Although the majority of the aortic arch abnormalities are physiological variants, it is crucial not to miss those lesions since they might require appropriate management, especially if clinically significant abnormalities such as aortic dissections or aneurysms are incidentally detected [11,12,13]. Unexpected aortic arch lesions have been observed in several previous studies. However, previous studies have mainly focused on the aortic arch abnormalities revealed using computed tomography (CT) [14,15,16,17]. It is not difficult to identify aortic arch abnormalities on neck CT angiography if the scan range is appropriate as the CT modality delivers clear and specific information. The previous studies which investigated incidental vascular findings by using MRI have mainly utilized brain MRI examinations [18,19]. To the best of our knowledge, no previous study has evaluated incidental aortic arch abnormalities that are detected on neck MR angiography. Therefore, this retrospective study aimed to investigate the prevalence of incidental aortic arch abnormalities and estimate the potential clinical significance of aortic arch abnormalities as a blind spot identified on contrast-enhanced neck MR angiography.

## 2. Materials and Methods

### 2.1. Study Population

**Figure 1:**
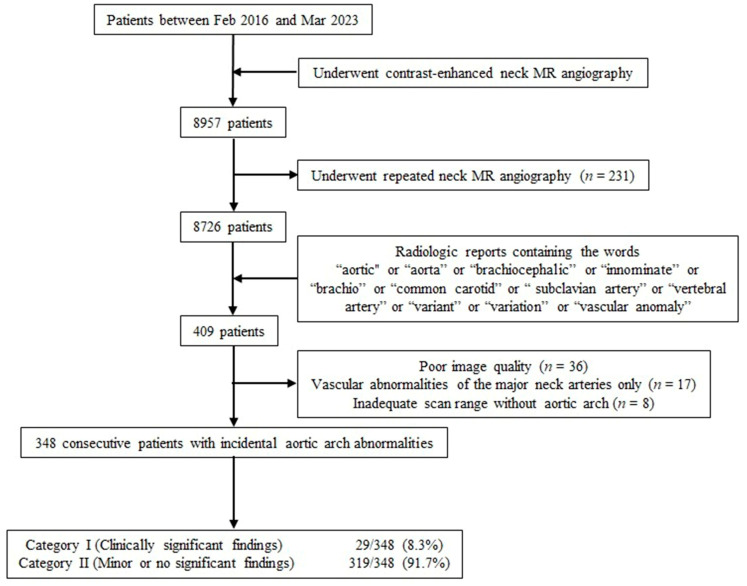
Flow diagram of the enrolled patients in this study.

A total of 8957 patients who underwent diagnostic contrast-enhanced neck MR angiography examinations between February 2016 and March 2023 were identified through a retrospective review of our institutional database. After excluding the data of 231 patients with repeated neck MR angiography images, the data of 8726 patients were used for this study. Afterward, we analyzed the data of 409 patients based on the radiological reports of neck MR angiography that mentioned the keywords “aortic”, “aorta”, “brachiocephalic”, “innominate”, “brachio”, “common carotid”, “subclavian artery”, “vertebral artery”, “variant”, “variation”, or “vascular anomaly”. Of the 409 patients, 61 patients were excluded due to poor image quality (36 patients), vascular abnormalities of the major neck arteries only (17 patients), and inadequate scan range, excluding the aortic arch (8 patients). Therefore, a total of 348 patients were finally included in this study (Figure 1). These patients included 175 (50.3%) males and 173 (49.7%) females (age range, 21–89 years; mean age, 63.6 ± 15.1 years). The study was approved by the Institutional Review Board of Gyeongsang National University Changwon Hospital (No. GNUCH 2023-03-039) on 5 April 2023. Neither patient consent nor informed consent were required due to the retrospective nature of the study.

### 2.2. Imaging Parameters of Contrast-Enhanced Neck MR Angiography

Contrast-enhanced neck MR angiography was performed using two different 3T MR scanners. Our contrast-enhanced neck MR angiography was obtained by utilizing the following parameters: repetition time (TR)/echo time (TE), 4.3/1.5 ms; flip angle, 25°; field of view (FOV), 340 × 283 mm; slice thickness, 1.4 mm; voxel size, 1.1 × 1.3 × 1.4 mm; no acceleration, bandwidth, 391 Hz/pixel; and acquisition time, 31 s for Signa™ Architect (GE Healthcare, Waukesha, WI, USA); TR/TE, 3.9/1.5 ms; flip angle, 27°; FOV, 320 × 270 mm; slice thickness, 1.2 mm; voxel size, 0.6 × 0.7 × 1.2 mm; acceleration factor (SENSE), phase (3) and slice (1); bandwidth, 744 Hz/pixel; and acquisition time, 38 s for Ingenia 3.0 CX (Philips Healthcare, Best, The Netherlands). The contrast material used for all the patients was 6–7.5 mL of Gadovist and the flow rate was set at 1.5 mL/s. Immediately after completion of the contrast agent injection, 30 mL of sterile isotonic 0.9% saline solution was injected at a rate of 2 mL/s using a power injector.

### 2.3. Imaging Analyses

The contrast-enhanced neck MR angiography images were reviewed by a faculty neuroradiologist (H.-J.B.) with 13 years of experience in evaluating incidental aortic arch abnormalities. The reader also assessed the presence of combined major intracranial and extracranial arterial abnormalities and additional imaging studies (i.e., brain MRI, brain MR angiography, or brain/neck CT or CTA) using the digital picture archiving and communication system (PACS). 

A faculty cardiothoracic radiologist (M.H.), with 3 years of experience, reviewed and categorized the aortic arch abnormalities with extracranial and intracranial arterial abnormalities based on clinical and laboratory findings and assessed the surgical or nonsurgical aortic treatment using electronic medical records. 

### 2.4. Statistical Analyses

Descriptive statistics were calculated, with the categorical variables presented as numbers with percentages, and continuous variables expressed as mean ± standard deviations (SD). The chi-square test and Fisher’s exact test were used for group comparisons of categorical variables according to the presence and clinical significance of the aortic abnormalities and major intracranial and extracranial abnormalities related to the underlying aortic abnormalities. All statistical analyses were performed using a statistical software package (SPSS version 26.0, IBM, Armonk, NY, USA). The statistical significance was set at *p* < 0.05 (two-sided).

## 3. Results

### 3.1. Study Patients

Among the total of 348 consecutive patients with incidental aortic arch abnormalities, 12 individuals had two or more aortic arch abnormalities and 336 patients had just one aortic arch abnormality. Thus, there were 360 lesions with incidentally detected aortic arch abnormalities on contrast-enhanced neck MR angiography in the studied patients.

### 3.2. Incidental Aortic Arch Abnormalities on Contrast-Enhanced Neck MR Angiography

Incidental aortic arch abnormalities can be summarized into seven types as demonstrated in Table 1. By referring to and modifying a previous study [3], we classified all aortic abnormalities into two categories according to their clinical significance: Category I represents clinically significant findings that include aortic aneurysm and aortic dissection which can affect the patient’s clinical course. These lesions require additional imaging studies to achieve accurate diagnoses and appropriate management. Category II, on the other hand, represents minor or no clinically significant findings that do not require further diagnostic studies or significant therapeutic processes. 

We have summarized the incidental aortic arch abnormalities in Table 1. Of the 348 patients, 29 patients (8.3%) were classified as Category I whereas the other 319 patients (91.7%) were classified as Category II. There were 22 aortic dissections (6.3%) and 7 aortic aneurysms (2.0%), which were included as Category I lesions.

Among the 22 aortic dissection cases, 17 patients had newly diagnosed aortic dissections, which were detected on neck MR angiography because they underwent brain MRI, intracranial time-of-flight (TOF) MR angiography, and neck MR angiography to evaluate their neurological deficits. By contrast, the other five patients were already aware of their diagnosis at the time of neck MR angiography. Of the 22 patients with aortic dissection, 14 patients with Stanford type A dissection (63.6%) underwent aortic surgery at our hospital, which included hemiarch replacement (11/14, 78.6%), total arch replacement (2/14, 14.3%), and thoracic endovascular aortic repair (TEVAR, 1/14, 7.1%).

Among the seven patients with aortic aneurysms, five patients were newly diagnosed with aortic aneurysms on neck MR angiography. These included the two patients diagnosed during medical check-ups. The other two patients had been diagnosed with aortic aneurysms before their neck MR angiography examinations. Of the seven aortic aneurysm cases, two patients (28.6%) underwent aortic surgery at our hospital; and one patient (14.3%) underwent TEVAR.

### 3.3. Intracranial and Extracranial Arterial Abnormalities beyond the Aortic Arch on Contrast-Enhanced Neck MR Angiography

We described the details of coexisting intracranial and extracranial arterial abnormalities on contrast-enhanced MR angiography and classified them into eight different kinds of abnormalities, as demonstrated in Table 2. Each abnormality was also divided into two categories according to their clinical significance similar to the categorization of the aortic arch abnormalities: Category I represents the clinically significant findings while Category II represents minor or no clinically significant findings. 

Of the 348 patients, 250 patients (71.8%) had intracranial abnormalities. Among these 250 patients, Category I comprised 130 lesions (52.0%). These lesions included 37 severe stenoses (14.8%), 23 occlusions (9.2%), 66 aneurysms (26.4%), and 4 arteriovenous malformations (1.6%). Category II comprised 232 lesions (92.8%), including 106 lesions with mild to moderate stenoses (42.4%), 18 penetrating atherosclerotic ulcers (7.2%), 17 junctional dilatations (6.8%), 75 congenital anomalies (30%), and 16 stent placements (6.4%).

In addition, 136 patients (348/136, 39%) had extracranial arterial abnormalities. Of these 136 patients, Category I comprised 38 lesions (27.9%), including 23 severe stenoses (16.9%), 10 occlusions (7.4%), and 5 aneurysms (3.7%). Category II comprised 112 lesions (82.4%), including 94 lesions with mild to moderate stenoses (69.1%), 8 penetrating atherosclerotic ulcers (5.9%), 2 junctional dilatations (1.5%), 7 congenital anomalies (5.1%), and 1 stent placement (0.7%).

### 3.4. Comparison between the Aortic Arch Abnormalities and Coexisting Supra-Aortic Arterial Abnormalities According to Clinical Significance

**Figure 2:**
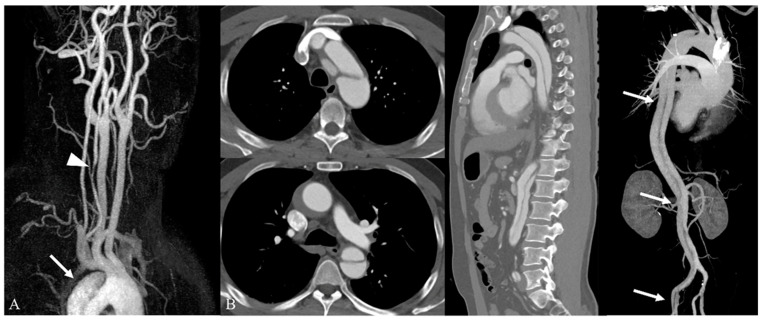
Incidentally detected aortic dissection (Stanford type A) during work-up for dizziness and left arm weakness. (**A**) In contrast-enhanced neck MR angiography (3D MIP image), there is a double-lumen lesion with intimal flap (arrow), suggesting true and false lumens in the mid to distal aortic arch involving the proximal descending aorta within the scan range. Coexisting focal stenosis is also noted in the left vertebral artery, V2 segment (arrowhead); (**B**) in contrast-enhanced aorta CT, the Stanford type A aortic dissection involves the whole aorta and extends to the left common and left external iliac arteries (arrows).

**Figure 3:**
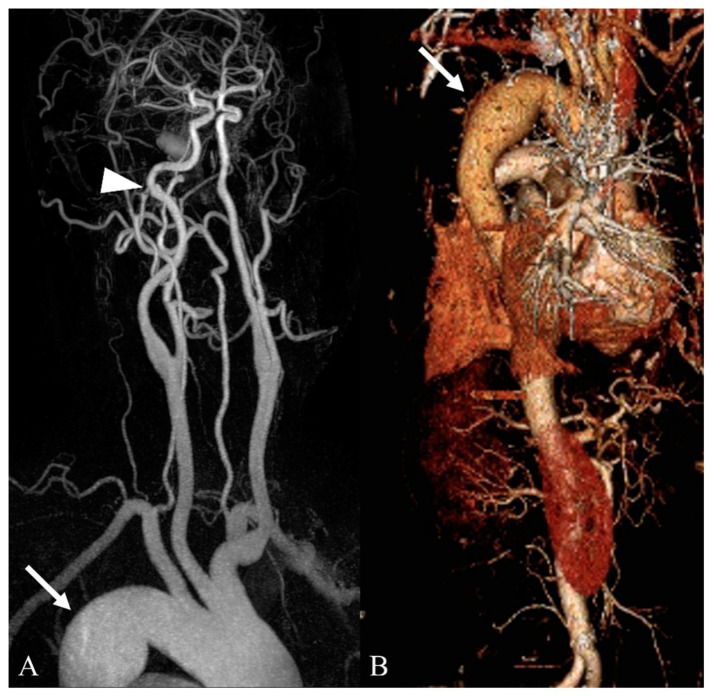
Incidentally detected aortic aneurysm during work-up for a headache in a patient with renal cell carcinoma. (**A**) In contrast-enhanced neck MR angiography (3D MIP image), there is a saccular aneurysm in the distal aortic arch (arrow). In the left distal internal carotid artery (C1 segment), a small bulging lesion is noted with a flap-like lesion, suggesting focal dissection (arrowhead). (**B**) In contrast-enhanced aorta CT, the aortic aneurysm is clearly depicted (arrow).

**Figure 4:**
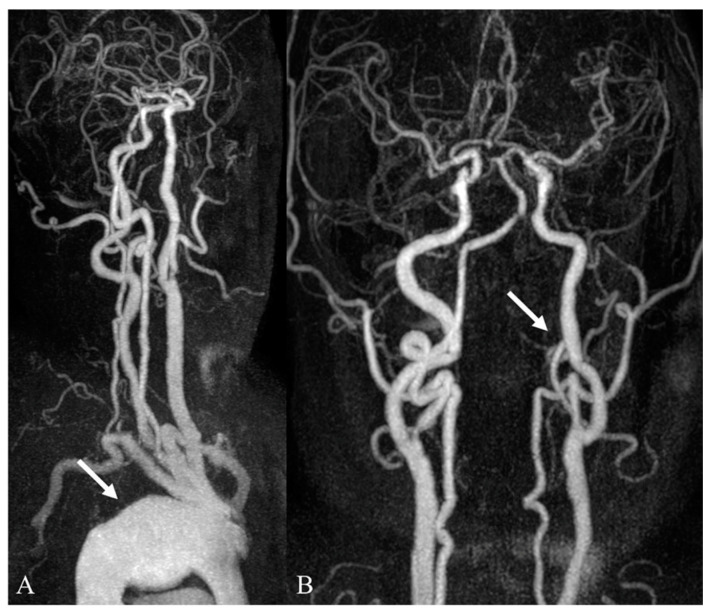
Incidentally detected aortic aneurysm during work-up for a transient ischemic attack. (**A**,**B**) In the 3D MIP images of contrast-enhanced neck MR angiography, there is a saccular aneurysm in the aortic arch (arrow in (**A**)). There is a coexisting segmental occlusion of the left vertebral artery, V4 segment (arrow in (**B**)).

**Figure 5:**
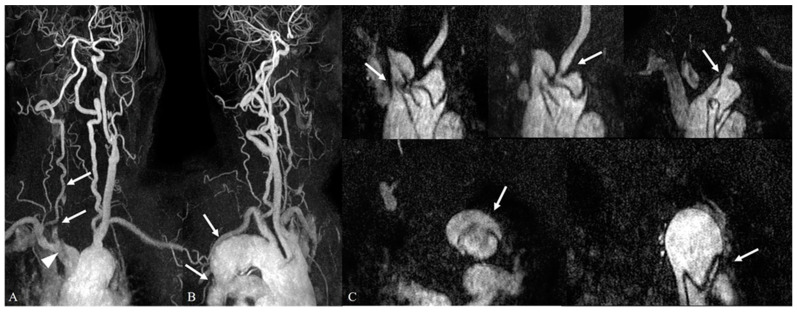
Incidentally detected aortic dissection (Stanford type A) during work-up for altered mental status. (**A**,**B**) In the 3D MIP images of contrast-enhanced neck MR angiography, there is no contrast opacification of the right common carotid, right internal carotid, and right external carotid arteries, suggesting occlusion (arrows in (**A**)). There is an intimal flap in the distal brachiocephalic artery near the origin of the right common carotid artery, suggesting arterial dissection (arrowhead in (**A**). Although the aortic arch demonstrates irregular features, there is no definite evidence of dissection (**B**). (**C**) In dynamic source images of contrast-enhanced neck MRA, the Stanford type A aortic dissection is detected along the entire aortic arch. The dissection involves the origin sites of the major neck arteries, including the brachiocephalic artery, right common carotid artery, left common carotid artery, and left subclavian artery (arrows in the upper row). Moreover, it extends to the descending aorta distally (arrows in the lower row).

Of the 29 patients with Category I aortic arch abnormalities, 24 patients (82.7%) demonstrated coexisting arterial abnormalities beyond the aortic arch (Figure 2, Figure 3, Figure 4 and Figure 5). However, 259 of the 319 patients (81.2%) with Category II aortic arch abnormalities showed coexisting non-aortic arterial abnormalities (Table 3).

The coexisting non-aortic arterial abnormalities were divided into two categories according to their clinical significance to evaluate the clinical relevance of the aortic arch abnormalities. There was a significantly higher prevalence of Category I aortic arch abnormalities (13/29, 44.8%) in the patients with Category I coexisting non-aortic arterial abnormalities than that of Category II aortic arch abnormalities (87/319, 27.3%) (*p* = 0.044, Table 3).

### 3.5. Comparison of Location-Specific Coexisting Arterial Abnormalities with Aortic Arch Abnormalities According to Clinical Significance

Table 4 demonstrates the comparison of location-specific coexisting arterial abnormalities and aortic arch abnormalities according to their clinical significance. Among the 29 patients with Category I aortic arch abnormalities, 21 patients (72.4%) had intracranial arterial abnormalities, 12 patients (41.4%) had extracranial abnormalities, and 9 patients had both intracranial and extracranial abnormalities. Among those with Category II aortic arch abnormalities, 229 patients (72.0%) had intracranial arterial abnormalities, 124 patients (28.9%) had extracranial abnormalities, and 94 patients had both intracranial and extracranial abnormalities. 

The presence of Category I intracranial arterial abnormalities was associated with a higher rate of Category I aortic arch abnormalities (31.0%) than that of Category II aortic arch abnormalities (19.4%); however, the difference was not statistically significant (*p* = 0.138). The aortic arch abnormalities demonstrated a higher tendency for Category I aortic arch abnormalities (17.2%) than for Category II aortic arch abnormalities (7.8%), without statistical significance (*p* = 0.136).

## 4. Discussion

Due to the increasing use of neck MR angiography for diagnosing various intracranial and extracranial illnesses, the likelihood of observing unintended results, such as aortic arch abnormalities inside the radiological scan range, has increased [7]. These incidental findings of aortic arch abnormalities detected on neck MR angiography can often be a diagnostic challenge for radiologists because of the limitations associated with the handling of incidental lesions detected on neck MR angiography. These can also result in an anatomical blind spot due to the focus on assessing the origin sites and entire courses of major neck arteries in the scan range, inadequate aortic arch coverage caused by scan range variations between hospitals, patients’ respiratory or motion artifacts, and lack of synchronous dedicated additional sequences for the incidental aortic arch abnormalities during the examinations [8,9].

The reported prevalence of unexpected aortic arch lesions on cross-sectional imaging ranges between 0.4% and 8%. However, all these lesions represent a fraction of the aortic dissections or aneurysms detected on CT examinations [14,15]. The rates of incidentally discovered aortic dissections or aneurysms in the present study were 2.0% and 6.3%, respectively. This is similar to that reported in previous studies even though our study evaluated neck MR angiography as an imaging modality. To the best of our knowledge, no previous studies have evaluated aortic arch abnormalities incidentally detected on neck MR angiography or analyzed aortic arch lesions other than aortic dissections or aneurysms, which were divided according to clinical significance. Therefore, we retrospectively assessed the prevalence and clinical implications of incidental aortic arch abnormalities as blind spots detected on contrast-enhanced neck MR angiography.

In the present study, the prevalence of severe stenosis or occlusion in the intracranial and extracranial systems was 14.8% or 9.2% and 16.9% or 7.4%, respectively, which is similar to the results of previous studies [20,21]. However, the prevalence of intracranial and extracranial aneurysms was 26.4% and 3.7%, respectively. This finding is inconsistent with previous studies, which reported a prevalence of intracranial aneurysms of up to 2.8% [19,22]. In addition, 232 patients (232/250, 92.8%) had Category II abnormalities in the intracranial arteries, whereas 112 patients (112/136, 82.4%) had Category II abnormalities in the extracranial arteries. These findings are also inconsistent with the results of previous studies, which reported rates of abnormalities of up to 60% [23,24]. The reason for these discrepancies is as yet unclear; however, they could be related to the selection bias in this retrospective study, and differences in imaging modalities, scanning parameters, or scan range. 

In the present study, the prevalence of abnormalities with high clinical importance in the aortic arch when there was also an abnormality in the intracranial or extracranial artery was 31.0% and 19.4%, respectively, which is higher than those in the other groups, despite not being statistically significant (*p* = 0.138 vs. 0.136, respectively). However, considering the relationship between the coexisting arterial abnormalities and aortic arch abnormalities, the prevalence of developing an abnormality with high clinical importance in the aortic arch was 44.8% when the patients had comorbid arteries with high clinical importance, demonstrating a higher tendency than the other group (27.3%). Conversely, if the accompanying arterial abnormalities were minor or had no clinically significant findings, the prevalence of observing minor or no significant findings in the scanned aortic arch was 53.9%, which was higher than that of the other group (37.9%), and also statistically significant (*p* = 0.044). Our results were in line with some previous studies which revealed that individuals with severe arterial stenosis in one particular artery bed were more likely to experience a clinical condition caused by severe arterial stenosis at another site [25,26]. Therefore, it is suggested that patients with intracranial arterial disease have a higher incidence of concomitant systemic arterial disease. This could explain the results with the simultaneous involvement of the aorta.

This study has several limitations that should be considered when interpreting the results. First, this study had a retrospective design, which suggests an inevitable selection bias. We included the study patients by reviewing the radiological records. Therefore, it is difficult to completely exclude the possibility of aortic arch abnormalities on neck MR angiography being overlooked by radiologists during the initial imaging evaluation. Accordingly, the actual prevalence of incidental aortic arch abnormalities on neck MR angiography might be higher than the results reported in this study. Second, it was a single-center study, and the patient population may have had different pathogenic factors and underlying diseases from the patient populations of other institutions or hospitals. Third, the reporting rate might be specific for the aortic arch abnormalities in our hospital and other institutions might report different findings. Therefore, the findings of this study may not be readily generalized to represent real-world clinical practice for all hospitals, and the clinical significance of the lesions may also be limited. Lastly, routine neck MR angiography protocols vary among the different institutions, which can affect the detectability of aortic arch lesions. Therefore, further studies with a larger sample size using different protocols of neck MR angiography and different MR systems can facilitate the validation of our findings and propose a lesion-specific diagnostic workflow using rational and cost-effective strategies for the management of incidental aortic arch abnormalities detected on neck MR angiography.

## 5. Conclusions

In the present study, aortic arch abnormalities with potential clinical significance incidentally detected on neck MR angiography exhibit a low prevalence of 8.3%. However, there was a statistically significant, clinically relevant association between aortic arch abnormalities and coexisting supra-aortic arterial abnormalities. Thus, the findings of the present study could be useful in understanding incidental aortic arch lesions detected on neck MR angiography. We also highlight the importance of clinical awareness for radiologists with respect to incidental aortic arch lesions as it can lead to accurate diagnosis and appropriate management through additional imaging work-up. Nevertheless, clinicians should keep in mind that there are possible limitations related to reproducibility and repeatability when obtaining neck MR angiography in different institutions with different protocols or different MR systems in real-world practice.

## Figures and Tables

**Table 1:** Summary of the incidental aortic arch abnormalities on contrast-enhanced neck MR angiography.

Aortic Arch Abnormalities	Number of Patients (%)	Number of Lesions (%)
Category I (Clinically significant findings)	29/348 (8.3%)	29/348 (8.3%)
Aortic dissection		22/348 (6.3%)
Aortic aneurysm		7/348 (2.0%)
Category II (Minor or no significant findings)	319/348 (91.7%)	331/348 (95.1%)
Direct aortic arch origin of vertebral artery		280/348 (80.4%)
Aberrant right subclavian artery		39/348 (11.2%)
Common trunk		8/348 (2.3%)
Right-sided aortic arch		4/348 (1.2%)

Note—Categories I and II were determined according to clinical significance. The data are presented with percentages in parentheses according to the raw numbers of patients and lesions.

**Table 2:** Intracranial and extracranial arterial abnormalities beyond the aortic arch on contrast-enhanced neck MR angiography.

Arterial Abnormalities	Intracranial Abnormality (250/348, 71.8%)	Extracranial Abnormality (136/348, 39%)
Category I (Clinically significant findings)	130/250 (52.0%)	38/136 (27.9%)
Severe stenosis	37/250 (14.8%)	23/136 (16.9%)
Occlusion	23/250 (9.2%)	10/136 (7.4%)
Aneurysm	66/250 (26.4%)	5/136 (3.7%)
Arteriovenous malformation	4/250 (1.6%)	NA
Category II (Minor or no significant findings)	232/250 (92.8%)	112/136 (82.4%)
Mild to moderate stenosis	106/250 (42.4%)	94/136 (69.1%)
Penetrating atherosclerotic ulcer	18/250 (7.2%)	8/136 (5.9%)
Junctional dilatation	17/250 (6.8%)	2/136 (1.5%)
Congenital anomaly	75/250 (30%)	7/136 (5.1%)
Stent placement	16/250 (6.4%)	1/136 (0.7%)

Note—Categories I and II were determined according to clinical significance. The data are presented as raw numbers of lesions with percentages in parentheses due to overlapping arterial abnormalities. NA, not applicable.

**Table 3:** Comparison between the aortic arch abnormalities and coexisting supra-aortic arterial abnormalities according to clinical significance.

	Aortic Arch Abnormalities		*p*-Value
	Category I (Clinically Significant Findings)	Category II (Minor or No Significant Findings)	
**Coexisting supra-aortic arterial** **abnormalities**	24/29 (82.7%)	259/319 (81.2%)	NA
Category I (Clinically significant findings) Category II (Minor or no significant findings)	13/29 (44.8%)	87/319 (27.3%)	0.044
	11/29 (37.9%)	172/319 (53.9%)	

Note—Categories I and II were determined according to clinical significance. The data are presented as raw numbers of patients with percentages in parentheses. NA, not applicable.

**Table 4:** Comparison of location-specific coexisting arterial abnormalities with aortic arch abnormalities according to clinical significance.

	Aortic Arch Abnormalities		*p*-Value
	Category I (Clinically Significant Findings)	Category II (Minor or No Significant Findings)	
**Intracranial arterial abnormalities**	21/29 (72.4%)	229/319 (72.0%)	NA
Category I (Clinically significant findings) Category II (Minor or no significant findings)	9/29 (31.0%)	62/319 (19.4%)	0.138
	12/29 (41.4%)	167/319 (52.4%)	
**Extracranial arterial abnormalities**	12/29 (41.4%)	124/319 (38.9%)	NA
Category I (Clinically significant findings) Category II (Minor or no significant findings)	5/29 (17.2%)	25/319 (7.8%)	0.136
	7/29 (24.1%)	99/319 (31.0%)	

Note—Categories I and II were determined according to clinical significance. Data are presented as raw numbers of patients with percentages in parentheses. NA, not applicable.

## Data Availability

Not applicable.
